# Persistent global marine euxinia in the early Silurian

**DOI:** 10.1038/s41467-020-15400-y

**Published:** 2020-04-14

**Authors:** Richard G. Stockey, Devon B. Cole, Noah J. Planavsky, David K. Loydell, Jiří Frýda, Erik A. Sperling

**Affiliations:** 1 Stanford University, Department of Geological Sciences, Stanford, CA 94305 USA; 2 School of Earth & Atmospheric Sciences, Georgia Institute of Technology, Atlanta, GA 30332 USA; 3 Department of Geology and Geophysics, Yale University, New Haven, CT 06511 USA; 4 School of the Environment, Geography and Geosciences, University of Portsmouth, Portsmouth, PO1 3QL UK; 5 Faculty of Environmental Sciences, Czech University of Life Sciences Prague, Prague, Czech Republic

**Keywords:** Element cycles, Element cycles, Palaeoceanography

## Abstract

The second pulse of the Late Ordovician mass extinction occurred around the Hirnantian-Rhuddanian boundary (~444 Ma) and has been correlated with expanded marine anoxia lasting into the earliest Silurian. Characterization of the Hirnantian ocean anoxic event has focused on the onset of anoxia, with global reconstructions based on carbonate δ^238^U modeling. However, there have been limited attempts to quantify uncertainty in metal isotope mass balance approaches. Here, we probabilistically evaluate coupled metal isotopes and sedimentary archives to increase constraint. We present iron speciation, metal concentration, δ^98^Mo and δ^238^U measurements of Rhuddanian black shales from the Murzuq Basin, Libya. We evaluate these data (and published carbonate δ^238^U data) with a coupled stochastic mass balance model. Combined statistical analysis of metal isotopes and sedimentary sinks provides uncertainty-bounded constraints on the intensity of Hirnantian-Rhuddanian euxinia. This work extends the duration of anoxia to >3 Myrs – notably longer than well-studied Mesozoic ocean anoxic events.

## Abstract (web summary)

The Late Ordovician mass extinction has been attributed to extended marine anoxia. Here, the authors use a metal isotope mass balance model and find the marine anoxic event lasted over 3 million years, notably longer than the anoxic event associated with the Permian-Triassic extinction and Cretaceous ocean anoxic events.

## Introduction

The early Silurian Rhuddanian Age directly follows the Hirnantian glaciation and Late Ordovician mass extinction^1,2^. Although biotic turnover may have begun in the Katian^3^, two extinction pulses are commonly considered to define the Late Ordovician mass extinction^2,4^: the first, around the Katian-Hirnantian boundary, is broadly attributed to global cooling and predominantly affected deep-water fauna^5^. The second, around the Hirnantian-Rhuddanian (Ordovician-Silurian) boundary, impacted animals throughout the water column^2^ and has been linked to a sulfide-dominated Hirnantian ocean anoxic event^6–8^. The Rhuddanian Age was an interval of sustained low marine diversity^3^, with the fossil plankton (graptoloid) record indicating a dramatic increase in average extinction rates from the Late Ordovician^9,10^ and benthic brachiopods exhibiting protracted post-extinction recovery^11,12^.

Geochemical data supporting the expansion of euxinic (anoxic, sulfide-rich) conditions are increasingly linked to extinction intensity around the Hirnantian-Rhuddanian boundary. A shift in the global marine redox landscape has been inferred from the extrapolation of regional iron and sulfur records^6,7^, and mass balance modeling of carbonate uranium isotope data^8^. It has also long been noted that there is an abundance of black shales in the early Silurian^13^ and the Rhuddanian stands out as an especially productive interval in the distribution of organic-rich mudrocks throughout the Phanerozoic^14^. Although this lithological transition has been linked to rapid post-glacial warming and marine anoxia in the early Silurian^13,15,16^, the negative shift in carbonate uranium isotopes (indicating the expansion of bottom-water euxinia) precedes sequence stratigraphic and geochemical evidence for a major glacial pulse in the Late Ordovician^1,8,17^. Whether the low-diversity interval and extended organic-rich shale deposition through the Rhuddanian Age is linked to an extended Hirnantian-Rhuddanian ocean anoxic event, persisting through glacial extremes and Rhuddanian deglaciation, therefore remains an open question.

Existing quantitative constraints on Hirnantian-Rhuddanian global marine anoxia have been based on a commonly used two-sink uranium isotope mass balance modeling framework with fixed flux and fractionation parameters^8^. There is increasing consensus that ferruginous (anoxic, sulfide-poor) conditions were common in the early Paleozoic^18^, although understanding of the implications of these depositional environments for trace metal isotope cycling remains limited. Separating non-euxinic and euxinic anoxic sinks in the δ^238^U mass balance significantly impacts model reconstructions of the global seafloor redox landscape^19^, as do varying fluxes and fractionations within reasonable limits based on observations from modern marine environments^20^. The combined effects of small deviations from commonly used model assumptions has the potential to dramatically alter implications for ancient marine environments. Therefore, moving forward, there is an obvious need for both improved statistical treatment of uncertainty and the use of multiple globally representative redox proxies and/or sedimentary archives in trace metal isotope mass balance models. With respect to the Hirnantian-Rhuddanian ocean anoxic event, both the complete duration and uncertainty-bounded estimates of the intensity and biogeochemical nature of the event are yet to be fully constrained.

In this study, we investigate the extent, nature, and duration of Hirnantian-Rhuddanian euxinia, and associated correlations with marine biodiversity and organic-rich shale deposition. Iron speciation measurements are used to establish the local water column conditions under which the Rhuddanian black shale succession from the Tanezzuft Shale Formation of the E1-NC174 core, Murzuq Basin, Libya, was deposited. Shale molybdenum and uranium concentrations are evaluated in the context of organic carbon correlations to infer the global applicability of measured trace metal isotope geochemistry. Molybdenum (δ^98^Mo) and uranium (δ^238^U) stable isotope data from the E1-NC174 core are then evaluated using a coupled Monte Carlo global mass balance model, in combination with previously generated carbonate δ^238^U data^8^. Our improved statistical treatment of uncertainties in metal mass balances, and incorporation of multiple trace metal isotopes and sedimentary archives, allows us to improve quantitative constraint on the spatial and temporal dynamics of Rhuddanian ocean euxinia (and associated uncertainties) during the Hirnantian-Rhuddanian ocean anoxic event. We confidently show that euxinic bottom-waters were likely two orders of magnitude more widespread than today for a period of >3 Myrs following the second pulse of the Late Ordovician mass extinction, significantly longer than well-studied Mesozoic ocean anoxic events.

## Results

### Geologic setting

**Fig. 1:**
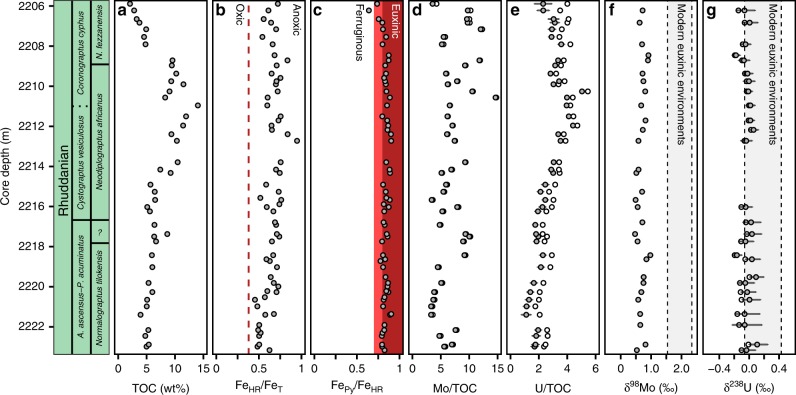
Geochemistry of the E1-NC174 core. Core depth is presented alongside the global and regional Rhuddanian graptolite biozones based on Loydell^21,22^. **a** Total organic carbon concentrations (TOC) are presented from Loydell et al.^25^. **b** Iron speciation highly reactive to total iron ratios; samples with Fe_HR_/Fe_T_ > 0.38 (dashed red line) are interpreted as anoxic. **c** Iron speciation pyrite to highly reactive iron ratios; samples with Fe_Py_/Fe_HR_ > 0.7–0.8 are interpreted as euxinic (red box, lighter red=possibly euxinic). **d** Mo concentrations normalized to total organic carbon concentrations (ppm/wt%). **e** U concentrations normalized to total organic carbon concentrations (ppm/wt%). **f** δ^98^Mo illustrated relative to modern euxinic shale range^47^. **g** δ^238^U illustrated relative to observed modern euxinic/anoxic shale range^44^. In Mo and U concentration and stable isotope plots, white points represent bulk measurements, and gray points represent measurements corrected for detrital input (for molybdenum, the difference between these values is often negligible). Error bars associated with trace metal concentrations illustrate 2 SD uncertainty on crustal concentrations used in detrital corrections. Error bars associated with trace metal isotopes illustrate combined analytical error (2 SE) and 2 SD uncertainty on crustal concentrations used in detrital corrections. Computed error bars for δ^98^Mo (Supplementary Data 1) are generally smaller than the data points illustrated here.

The E1-NC174 core records approximately three million years of Rhuddanian black shale deposition through the Tanezzuft Shale Formation of the Murzuq Basin, Libya^21–23^. The Murzuq Basin is an intracratonic depression that is believed to have originally extended northward across the passive margin of Gondwana^24^ (Supplementary Fig. 1), recording Cambrian through Silurian siliciclastic marine sedimentation. The economic importance of the mid-Rhuddanian hot shale as a hydrocarbon source rock has led to the detailed biostratigraphic characterization of the E1-NC174 core^21,25,26^, indicating that the stratigraphic record presented here spans from at least the lowermost Rhuddanian (*Normalograptis tilokensis* Biozone in Gondwana, *Akidograptus ascensus* Biozone in global biostratigraphy) to the uppermost Rhuddanian (*Neodiplograptus fezzanensis* Biozone in Gondwana, *Coronograptus cyphus* Biozone in global biostratigraphy; Fig. 1; Supplementary Note 1). The mid-Rhuddanian hot shale varies in thickness across northern Gondwana, a trend that has been interpreted as the product of sea level change relative to post-glacial paleorelief^24^. Molybdenum and uranium enrichment factors covary in proportion with the modern seawater Mo-U ratio (Supplementary Fig. 2), and the concentrations of both elements correlate linearly with organic carbon concentrations (Supplementary Fig. 3). These trends suggest that, despite this inferred depositional relationship with paleobathymetry, changes in sea level did not markedly impact the resupply of molybdenum and uranium from the open ocean to the Murzuq Basin.

### Molybdenum and uranium isotopes as tracers of marine redox

Molybdenum and uranium stable isotope ratios (δ^98^Mo and δ^238^U) have been developed as tracers of global marine redox conditions and widely applied to the geologic record^27–29^. There are distinct isotopic fractionation factors associated with the incorporation of both elements into oxic vs. euxinic sedimentary sinks, meaning that the extent of euxinic seafloor exerts strong controls on the δ^98^Mo and δ^238^U compositions of seawater^30,31^. Molybdenum and uranium have modern residence times of >400 kyr in seawater^30,32^ and are therefore expected to be well-mixed on the timescales of ocean circulation, even during times of more widespread anoxia^33^. The heavy isotopic compositions of euxinic sedimentary sinks in both the molybdenum and uranium systems mean that seawater δ^98^Mo and δ^238^U values are predicted to decrease during periods of expanded euxinia. Euxinic shales track the seawater molybdenum and uranium isotopic composition, albeit with a predictable fractionation factor in most cases^30,31^. Increases in the areal extent of bottom-water anoxia (and, by extension, authigenic enrichments in marine sediments) further lead to drawdown of dissolved seawater molybdenum and uranium concentrations^34^, highlighting the value of mass balance models that fully couple seawater metal concentrations and isotope values in reconstructions of global redox (see Methods—Mo-U mass balance model). The redox-sensitivity of predictable authigenic enrichment differs between molybdenum and uranium^34^, specifically with respect to the high H_2_S concentrations required for quantitative molybdenum reduction^35^. We therefore propose that interpreting sedimentary records of both trace metal isotope systems in combination improves constraint on the biogeochemical nature of anoxic intervals, as does the combination of δ^238^U data from multiple sedimentary archives.

One of the appeals of the molybdenum and uranium proxies is that they can be easily translated into a quantitative framework to estimate the areal extent of euxinic conditions^8,27,29,36^. The global molybdenum and uranium mass balances are described by a series of ordinary differential equations relating the concentrations and isotopic compositions of molybdenum and uranium in seawater to relevant sources and sinks (Methods—Mo-U mass balance model). In contrast to the modern ocean, ferruginous (anoxic, sulfide-free) bottom-waters are thought to have been prevalent in the early Paleozoic^18^. Consequently, both trace metal cycles are modeled here with three redox-sensitive sinks: oxic, euxinic, and a broadly defined ‘reducing’ sink intended to capture the behavior of ferruginous, as well as traditionally suboxic, settings (following Reinhard et al.^36^). The fluxes and isotopic fractionations associated with the trace metal sources and sinks in mass balance models are based on observations of trace metal behavior in modern environments^36–38^. We propose that the fixed parameterization employed in conventional mass balance approaches, although useful in quantifying general trends, inherently underrepresents uncertainty in the implications of trace metal cycling for global marine redox. To accommodate variations in trace metal behavior between modern depositional environments, and corresponding uncertainty about trace metal cycling in deep time, we employ Monte Carlo simulations, coupling the global molybdenum and uranium isotope mass balances. We randomly subsampled all model parameters that are known to vary across modern environments between minimum and maximum observed values (see Table 1 for parameter space and justification of parameter ranges). For all parameters, we assumed a uniform distribution, rather than forcing a most likely scenario with normal distributions. Broadly reducing, specifically ferruginous, environments are proposed to create some of the biggest uncertainties in this mass balance approach. We therefore widely varied the extent of broadly reducing environments for each logarithmically scaled scenario of ocean euxinia, between 0% of the global ocean floor and the remaining (non-euxinic) ocean floor available. Owing to the scarcity of modern ferruginous analogs^39^, we applied widely bounded fractionation factors for the broadly reducing sinks to incorporate plausible fractionation factors not observed in modern suboxic environments but expected based on laboratory experiments^40^. Supplementary Fig. 6 illustrates that alternative parameterizations of these fractionation factors do not significantly alter the results of the fully coupled δ^238^U_eux_ **+** δ^238^U_carb_ **+** δ^98^Mo_eux_ model.

The effects of expanding anoxic regions on local trace metal burial are also incorporated into our model using a pseudospatial scaling algorithm^36^. Estimates of trace metal burial in euxinic settings are conventionally based on observations from relatively shallow modern environments^37,38^. As widespread euxinia develops at a global oceanographic scale, authigenic enrichments in reducing environments occur at increasing depth as a physical consequence of expanded low-oxygen conditions. Trace metal complexes therefore travel greater distances through the water column, and are increasingly susceptible to remineralization^36^. The scaling factor used here links trace metal accumulation to organic carbon transport^41,42^ to provide first-order accommodation of the issues associated with using continental shelf settings to model deep-water trace metal accumulation under expanded reducing conditions. Local trace metal isotope fractionations are subsampled independently of global means to incorporate expected natural deviations of local depositional environments from global averages (Table 1). Full model equations are described in Methods—Mo-U mass balance model.

### Tracking local water column geochemistry

All of the black shale samples analyzed have Fe_HR_/Fe_T_ values > 0.4, and almost all have Fe_Py_/Fe_HR_ ratios above 0.7 (Fig. 1; 58 of 59 samples; Fe_Py_/Fe_HR_ **>** 0.8 for 49 of 59 samples). Comparing these ratios to empirically defined baselines^39^ indicates that the Tanezzuft black shale succession was deposited under persistently euxinic bottom-waters. These data confirm previous sedimentological inferences^25^ that the depositional environment of the E1-NC174 core was dominantly anoxic, and demonstrate the presence of free hydrogen sulfide (euxinia). Molybdenum and uranium concentrations are consistently enriched relative to average shale values (Supplementary Fig. 4), supporting inferences that bottom-waters were strongly reducing^34^. The persistence of local euxinia indicates that sedimentary δ^98^Mo and δ^238^U signatures are reliable records of contemporaneous seawater isotope values^43,44^. Cross-plots of Mo versus U enrichment factors show a linear relationship consistent with modern seawater Mo-U ratios and do not indicate the influence of a particulate shuttle^45^ (Supplementary Fig. 2). Mo/TOC and U/TOC ratios (Fig. 1, Supplementary Fig. 3) exhibit no directional stratigraphic trend through the E1-NC174 core, indicating that increased enrichments in molybdenum and uranium through the hot shale interval (Supplementary Fig. 4) are likely a product of organic carbon loading^41,42,46^. Similar trends are observed in vanadium enrichments, but not in metals such as chromium that are more sensitive to detrital input^34^ (Supplementary Fig. 4).

### Modeling global marine redox conditions

Uranium and molybdenum isotope data exhibit low variance around isotopically light mean values (relative to modern euxinic shales) throughout the E1-NC174 core (Fig. 1). Molybdenum isotope fractionations in low-sulfide conditions are expected to be highly variable^35,47^, whereas in high-sulfide conditions (with extensive thiomolybdate formation) fractionations are expected to exhibit low variance with near quantitative capture of seawater δ^98^Mo. We therefore make a case that low variances in δ^98^Mo (mean **=** 0.69‰, SD **=** 0.13‰) and δ^238^U (mean **=** −0.02‰, SD **=** 0.07‰) values are further indications that molybdenum and uranium were reduced with consistent fractionations (or, in the case of molybdenum, likely no fractionation) from seawater under the locally euxinic conditions overlying the Rhuddanian black shales of the Murzuq Basin. The absence of a directional stratigraphic response in δ^98^Mo and δ^238^U to organic carbon loading (and likely associated sea level change^25^) is further evidence that the trace metal isotope compositions of the E1-NC174 core shales were not controlled by variation in local redox conditions and therefore reliably record global seawater signals. Furthermore, the offset (~0.5‰) between δ^238^U_eux_ data presented here and published δ^238^U_carb_ data^8^ (reported mean value **=** −0.45‰) for the early Rhuddanian is consistent with modern observations of the fractionation between shallow-water carbonates and euxinic sedimentary sinks^44,48^ (Supplementary Fig. 5), adding support that the Murzuq Basin shales and Anticosti Island carbonates both record global trace metal isotope signals. Long-term variations in the marine molybdenum and uranium budgets are expected to be primarily controlled by the relative extent of strongly reducing sedimentary sinks due to high metal accumulation rates^30,31,37^. Isotopically light δ^98^Mo and δ^238^U values (relative to modern euxinic shales) are therefore broadly interpreted as recording globally expansive anoxic, likely euxinic, bottom-water conditions throughout the Rhuddanian.

**Fig. 2:**
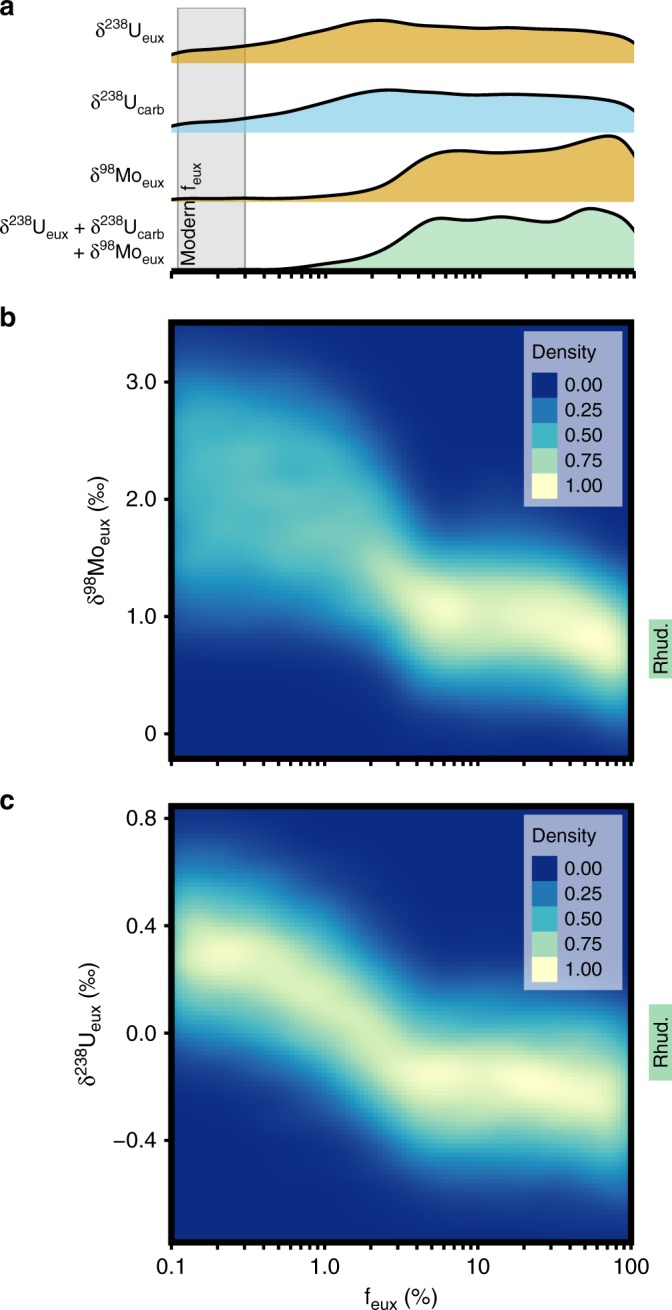
Estimates of euxinia (f_eux_) from Monte Carlo simulations. **a** Smoothed frequency distributions (upper panel) represent the distributions of f_eux_ scenarios compatible with the measured δ^238^U_eux_, δ^238^U_carb_^8^, δ^98^Mo_eux_, and combined carbonate^8^ and euxinic shale Mo-U isotope data (δ^238^U_eux_ + δ^238^U_carb_ + δ^98^Mo_eux_) (labeled, from top to bottom). The fully combined δ^238^U_eux_ + δ^238^U_carb_ + δ^98^Mo_eux_ distribution is taken as the best-constrained model of f_eux_ through this time interval. **b** Two-dimensional density plot shows the frequency distributions of modeled sedimentary δ^98^Mo_eux_, as calculated by global sensitivity analyses of the Mo isotope mass balance. **c** Two-dimensional density plot shows the frequency distributions of modeled sedimentary δ^238^U_eux_, as calculated by global sensitivity analyses of the U isotope mass balance. Green bars (right side of 2D density plots) illustrate the full range of Rhuddanian isotope data.

Molybdenum and uranium mass balance model results are illustrated as frequency distributions of predicted euxinic shale δ^98^Mo and δ^238^U values corresponding to a range of logarithmically scaled scenarios for global marine euxinia (Fig. 2, f_eux_ describes the fraction of global seafloor with euxinic bottom-waters). The two-dimensional density plots presented here are applicable to all intervals of geologic time with modern-style tectonic and weathering processes (see Supplementary Fig. 6 for the impact of elevated weathering rates), assuming that the molybdenum and uranium cycles are in steady-state and the trace metals are well-mixed in seawater. Smoothed frequency distributions in Fig. 2a illustrate the distributions of modeled f_eux_ values compatible with the range of measured euxinic shale values from the E1-NC174 core, and Rhuddanian shallow-water carbonates from Anticosti Island^8^. The corresponding f_eux_ ranges illustrate the implications of δ^238^U_eux_, δ^238^U_carb_ (from Bartlett et al.^8^), δ^98^Mo_eux_, and δ^238^U_eux_ **+** δ^238^U_carb_ **+** δ^98^Mo_eux_ values for the global extent of marine euxinia through the Rhuddanian Age. The combined δ^238^U_eux_ **+** δ^238^U_carb_ **+** δ^98^Mo_eux_ distribution is proposed as the best-constrained model of f_eux_ for this time interval, indicating that euxinic bottom-waters were most likely two orders of magnitude more widespread than modern (median f_eux_ estimate = 15.8%, mean = 29.2%), with a significant spread of feasible f_eux_ values (5th percentile=2.0%, 95th percentile = 100%). Despite these uncertainties, fully coupled Rhuddanian model results are not compatible with modern levels of ocean oxygenation (Fig. 2), and indicate modally different, dominantly euxinic ocean biogeochemistry throughout the Rhuddanian.

**Fig. 3:**
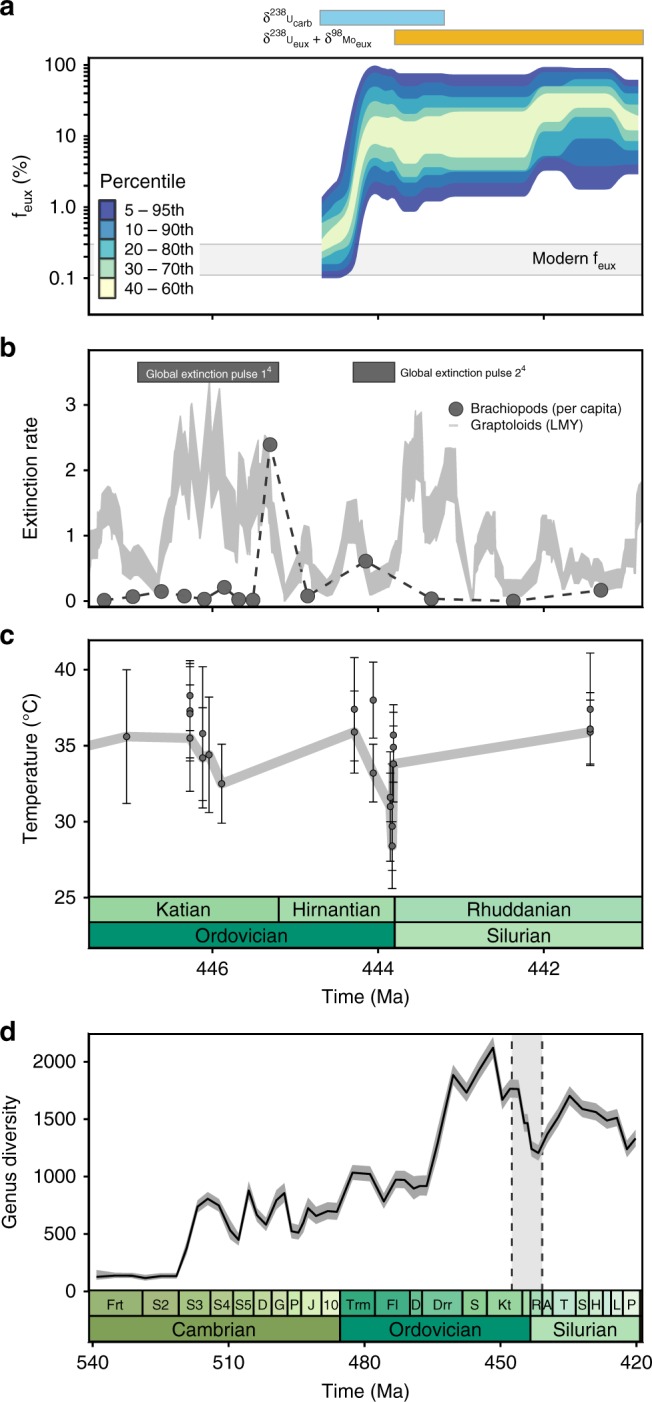
Ordovician-Silurian boundary environmental and biological change. **a** Modeled distribution of f_eux_ values across the Ordovician-Silurian boundary based on the geochemical data presented here and in Bartlett et al.^8^. Cross-validated LOESS models are fitted to percentile-bracketed estimates of global seafloor euxinia for each timestep (Methods—Mo-U mass balance model). The temporal extent of carbonate^8^ and euxinic shale records analyzed in this model framework are shown above. **b** Per-capita brachiopod extinction rates^5^ and per lineage million year (LMY) graptoloid extinction rates^9^ with extinction pulses from capture–recapture analyses^3,4^ schematically illustrated above. Envelope of graptoloid extinction rates illustrates mean values±68% quantiles from bootstrap means (1000 iterations) of median values for each 0.25 Myr time bin. We note that the relatively broad time bins of Kröger et al.^4^ leave the timing and duration of the first extinction pulse illustrated loosely constrained. **c** Sea-surface temperature estimates based on clumped isotope paleothermometry^1^—with a line fitted (as in Finnegan et al.^1^) to the lowest temperature estimates for each time bin. Error bars illustrate 1 SD analytical error reported in original study. **d** Genus-level diversity calculated by capture–recapture methods from the Paleobiology Database^3^, error envelope illustrates 95% confidence intervals for capture–recapture analyses. Vertical gray box illustrates time interval depicted in **a**–**c**, with the extended Cambrian–Silurian diversity trends illustrated as context for the end-Ordovician mass extinction and subsequent low-diversity interval. Age models are updated from original publications to reflect the Geologic Timescale 2012^23^ and illustrate stratigraphic relationships relative to δ^238^U_carb_ data^8^ where applicable (Methods—Stratigraphic age models; Supplementary Notes 2, 3).

The implications of the coupled Mo-U Monte Carlo mass balance model across the Ordovician-Silurian boundary are illustrated in Fig. 3a, based on Bartlett et al.^8^ and the new geochemical data presented in Fig. 1 (see Methods—Stratigraphic age models). The best-fit distribution is defined by cross-validated LOESS models fitted to percentiles of the distribution of f_eux_ estimates at each timestep. The time-dependent distribution is bounded by cross-validated LOESS models fitted to the 5th and 95th percentile f_eux_ estimates. These are considered the most representative (and conservative) estimate of uncertainty across the logarithmic range of marine redox scenarios investigated here. The onset of globally widespread euxinia modeled here is well correlated with the second pulse of the Late Ordovician mass extinction^2,4,5^ (Fig. 3), as indicated by the δ^238^U_carb_ data of Bartlett et al.^8^. Our model constrains uncertainty on the spatial extent of euxinic seafloor, demonstrating that under all parameterizations of the uranium isotope cycle this geochemical data requires a 1–2 order-of-magnitude change in seafloor redox landscape. Furthermore, the incorporation of a second redox-sensitive δ^238^U archive and δ^98^Mo data allow us to increase quantitative constraint on the extent of Rhuddanian euxinia (relative to δ^238^U_carb_ data alone, Fig. 2), and to extend the record of euxinia throughout the Rhuddanian Age.

## Discussion

The black shale trace metal isotope data, and model evaluation of new and published^8^ data, presented here, support previous inferences that the earliest Rhuddanian experienced widespread ocean anoxia^6–8^ and extends the duration of the Hirnantian ocean anoxic event by >2 million years to include the entire Rhuddanian Age (Fig. 3). Our coupled approach, using euxinic shale molybdenum and uranium isotope measurements in conjunction with existing carbonate δ^238^U data from the Hirnantian and lower Rhuddanian^8^, allows us to dramatically improve quantitative constraints on the intensity of global marine euxinia. Namely, previously published Rhuddanian δ^238^U_carb_ data^8^, when analyzed with a three sink redox model and full accounting of uncertainty in trace metal cycles, results in similar mean (17.9%) and median (6.3%) f_eux_ results to our fully coupled model, but with a 5th percentile at 0.3% (Fig. 2). This broad distribution of compatible f_eux_ estimates means that modeling of carbonate δ^238^U data alone cannot confidently reject f_eux_ estimates compatible with, or at least similar in magnitude to, modern anoxic seafloor^36,37^ for the Rhuddanian Age. A similar resulting f_eux_ distribution is obtained for our δ^238^U_eux_ data alone (Fig. 2), and these analyses echo other recent modeling results, demonstrating that the uranium isotope system in isolation has relatively limited resolution when all applicable redox sinks and sources of uncertainty are taken into account^19^. However, global sensitivity analyses demonstrate that for the combined δ^238^U_eux_ + δ^238^U_carb_ + δ^98^Mo_eux_ model, even with considerable deviations from modern molybdenum and uranium cycling, it is very unlikely that the isotopic record through the Rhuddanian could have been generated without strongly reducing depositional environments likely two orders of magnitude more extensive than today (Fig. 2). The multiproxy approach applied here therefore indicates that early Silurian marine redox dynamics were modally different to the modern ocean, and adds statistically robust support to previous inferences of Rhuddanian euxinia^8^. Furthermore, these extensively euxinic conditions were relatively stable for at least the duration of the Rhuddanian Age, extending the duration of the Hirnantian-Rhuddanian OAE by approximately three times (to at least three million years). The Hirnantian-Rhuddanian OAE therefore lasted significantly longer than well-studied Mesozoic ocean anoxic events^49–51^, and was more similar in duration to other Paleozoic anoxic intervals in the Cambrian^33^ and Devonian^52^.

The emerging picture of the Rhuddanian Age is one of poorly oxygenated oceans, with relatively low benthic marine biodiversity^3^ and enhanced organic carbon burial^13,14^. Recent models of extinction timing based on global database analyses^3,4^ (full curve shown in Fig. 3d, schematic representation of extinction pulses shown in Fig. 3b) add support to inferences that the second pulse of the Late Ordovician mass extinction coincided with the onset of the Hirnantian-Rhuddanian ocean anoxic event^8^ (Fig. 3). The capture–recapture analysis of early Paleozoic marine biodiversity^3^ illustrated in Fig. 3d is taken here as the most reliable reconstruction of both the extinction pulses and subsequent low-diversity interval owing to the explicit consideration of taphonomy and sampling bias, and the marked improvement in temporal resolution relative to previous reconstructions. High-resolution extinction rate analyses of brachiopod^5^ and graptoloid^9,10^ fossil occurrence data, although more vulnerable to sampling biases, also support a major extinction event that is well correlated with the onset of global euxinia (Fig. 3b). Post-extinction recovery appears to have been limited in the Rhuddanian, with the Hirnantian-Rhuddanian OAE as described in this study (Fig. 3a) constituting a relative low-point in Middle Ordovician through Silurian biodiversity^3^ (Fig. 3d). Global biodiversity estimates suggest that marine biodiversity did not reach pre-extinction levels for more than five million years^3^ (Fig. 3d). Specifically, Silurian brachiopod biodiversity shows a protracted post-extinction recovery, lasting until at least the Telychian^11,12,53^ (starting ~436 Ma), and high rates of planktonic (graptoloid) turnover persisted throughout the Silurian^9,10^ (Fig. 3b). The correlation between the second pulse of the Late Ordovician extinction^4^ and the prolonged low-diversity interval^3^ with the onset and persistence of widespread global marine euxinia indicates a major potential role for global ocean deoxygenation (and/or its external drivers) in governing global marine biodiversity.

The Rhuddanian trace metal isotope record presented in this study quantitatively supports existing observations of extended euxinic shale deposition from sedimentological compilations^13–15^. This euxinic interval corresponds not only to the North African hot shale source rocks, but to established gas shales such as the Longmaxi Formation in China^54^ and potential hydrocarbon targets such as the Bardo Formation in the Baltic Basin^55^. The inferred expansion of global marine euxinia is thus correlated with the global appearance of economically important black shales in the early Silurian^14^, which our work indicates were probably deposited over large swaths of the global seafloor. Although regional basin dynamics likely control specific differences in the hydrocarbon potential of Rhuddanian black shales, the analyses presented here demonstrate the governing role that global-scale ocean biogeochemistry can play in the distribution of organic-rich mudrocks.

The Hirnantian-Rhuddanian ocean anoxic event appears to be sustained across both peak Hirnantian glacial cooling^1,8^ and post-glacial Rhuddanian warming^8,13,15,16^. Therefore, although the onset of the anoxic event may coincide with a major pulse of the Hirnantian glaciation (cooccurring with the inception of Late Ordovician Glacial Cycle 3^8,17^, and predating the major late Hirnantian cooling episode indicated in oxygen isotope^8^ and clumped isotope^1^ data), the extended global record of Rhuddanian euxinia presented here demonstrates that the modally different ocean biogeochemistry we describe is non-specific to global climate state. The lack of correlation between model reconstructions of euxinia and both stratigraphic and geochemical reconstructions of climatic change indicate that this episode of ocean euxinia defies conventional, Cenozoic-Mesozoic models of temperature-driven marine euxinia^56^. The reducing post-extinction oceans described here also likely persisted much longer than more frequently discussed Cenozoic and Mesozoic deep-time analogs for ocean deoxygenation. In other words, the Hirnantian-Rhuddanian ocean anoxic ‘event’ was an extended state change in marine redox, lasting a minimum of approximately six times longer than Cretaceous OAE2, and approximately two times the duration of early Triassic anoxia, based on current geochronological models and proxy data^23,50,51^. This adds to the existing examples of extended-duration Paleozoic ‘OAEs’ in the Cambrian^33^ and Devonian^52^. Secular changes in atmospheric oxygen^57,58^ and the biological pump^57,59^, as well as feedbacks between global climate, tectonics, and ocean circulation^16,60^, are all plausible driving mechanisms for this difference in the nature of ocean anoxic events through the Phanerozoic. Statistically robust models of global marine redox based on multiple geochemical proxies and/or sedimentary archives, as presented here, will provide valuable constraint for approaches that seek to address this problem through combined ocean circulation and biogeochemical modeling.

In summary, the Tanezzuft Shale Formation of the Murzuq Basin, Libya, hosts a continuous record of persistently euxinic black shale deposition, constraining the succession’s exceptional suitability for the reconstruction of globally redox-sensitive trace metal isotope records. Combined Monte Carlo mass balance modeling of δ^238^U_eux_, δ^238^U_carb_, and δ^98^Mo_eux_ measurements support reconstructions of a Hirnantian ocean anoxic event continuing into the earliest Silurian *ascensus* Zone, and further demonstrate that extended marine euxinia then lasted throughout the Rhuddanian Age (likely >3 million years). Euxinic seafloor area can be confidently reconstructed as at least an order of magnitude, and more likely two orders of magnitude, more widespread in the early Silurian than the modern. Prolonged recovery from the Late Ordovician mass extinction supports the notion that ocean deoxygenation has repeatedly had prolonged impacts on marine ecosystems at the timescales of geological stages/ages. The early Silurian marine system appears to be defined by low benthic marine biodiversity, enhanced organic carbon burial, and dramatically expanded euxinic bottom-waters.

## Methods

### Mo-U mass balance model

The concentration and isotope values of molybdenum and uranium in seawater are described by Eqs. 1–4.

1 $$\frac{{{\mathrm{d}}[{\mathrm{Mo}}]_{{\mathrm{sw}}}}}{{{\mathrm{dt}}}} = {\mathrm{F}}_{{\mathrm{riv}}} - {\mathrm{F}}_{{\mathrm{oxic}}} - {\mathrm{F}}_{{\mathrm{red}}} - {\mathrm{F}}_{{\mathrm{eux}}}$$

2 $$\frac{{{\mathrm{d}}[{\mathrm{Mo}}]_{{\mathrm{sw}}}{\mathrm{\delta }}^{98}{\mathrm{Mo}}_{{\mathrm{sw}}}}}{{{\mathrm{dt}}}} =	 \, {\mathrm{F}}_{{\mathrm{riv}}}{\mathrm{\delta }}^{98}{\mathrm{Mo}}_{{\mathrm{riv}}} - {\mathrm{F}}_{{\mathrm{oxic}}}({\mathrm{\delta }}^{98}{\mathrm{Mo}}_{{\mathrm{sw}}} + \Delta ^{98}{\mathrm{Mo}}_{{\mathrm{oxic}}}) \\ 	- {\mathrm{F}}_{{\mathrm{red}}}({\mathrm{\delta }}^{98}{\mathrm{Mo}}_{{\mathrm{sw}}} + \Delta ^{98}{\mathrm{Mo}}_{{\mathrm{red}}}) - {\mathrm{F}}_{{\mathrm{eux}}}({\mathrm{\delta }}^{98}{\mathrm{Mo}}_{{\mathrm{sw}}} + \Delta ^{98}{\mathrm{Mo}}_{{\mathrm{eux}}})$$

3 $$\frac{{{\mathrm{d}}[{\mathrm{U}}]_{{\mathrm{sw}}}}}{{{\mathrm{dt}}}} = {\mathrm{F}}_{{\mathrm{riv}}} - {\mathrm{F}}_{{\mathrm{oxic}}} - {\mathrm{F}}_{{\mathrm{red}}} - {\mathrm{F}}_{{\mathrm{eux}}}$$

4 $$\frac{{{\mathrm{d}}\left[ {\mathrm{U}} \right]_{{\mathrm{sw}}}{\mathrm{\delta }}^{238}{\mathrm{U}}_{{\mathrm{sw}}}}}{{{\mathrm{dt}}}} =	 \, {\mathrm{F}}_{{\mathrm{riv}}}{\mathrm{\delta }}^{238}{\mathrm{U}}_{{\mathrm{riv}}} - {\mathrm{F}}_{{\mathrm{oxic}}}({\mathrm{\delta }}^{238}{\mathrm{U}}_{{\mathrm{sw}}} + \Delta ^{238}{\mathrm{U}}_{{\mathrm{oxic}}}) \\ 	- {\mathrm{F}}_{{\mathrm{red}}}({\mathrm{\delta }}^{238}{\mathrm{U}}_{{\mathrm{sw}}} + \Delta ^{238}{\mathrm{U}}_{{\mathrm{red}}}) - {\mathrm{F}}_{{\mathrm{eux}}}({\mathrm{\delta }}^{238}{\mathrm{U}}_{{\mathrm{sw}}} + \Delta ^{238}{\mathrm{U}}_{{\mathrm{eux}}})$$

Fluxes for redox-sensitive sinks are defined as

5 $${\mathrm{F}}_{i} = {\mathrm{b}}_{i}{\mathrm{A}}_{i}{\mathrm{\alpha }}_{i}\frac{{[{\mathrm{Me}}]_{{\mathrm{sw}}}}}{{[{\mathrm{Me}}]_{{\mathrm{M}}.{\mathrm{sw}}}}}$$

Where b_*i*_ represents per area burial flux, A_*i*_ is the seafloor area of the redox-sensitive sink, α_*i*_ is a pseudospatial scaling coefficient relating burial rate to the area of the sink^36^, [Me]_sw_ is the mean modeled concentration of the metal in seawater, and [Me]_M.sw_ is the mean modern concentration of the metal in seawater.

The area of the redox-sensitive sink is defined as

6 $${\mathrm{A}}_i = {\mathrm{Af}}_i$$

Where A is the total modern seafloor area, and f_*i*_ is the proportion of the global seafloor that the redox-sensitive sink covers.

In the Monte Carlo simulations, f_eux_ is modeled in 31 equally spaced, logarithmically scaled steps, with 1000 model runs per f_eux_ scenario. The areal extent of the broadly reducing sink, f_red_, is included in the global sensitivity analysis, and allowed to vary between 0 and 100-f_eux_%. The range of f_ox.lim_, the maximum extent of oxic deposition, is defined in Table 1 based on Reinhard et al.^36^ and physical oceanographic limits.

7 $${\mathrm{f}}_{{\mathrm{oxic}}} = {\mathrm{f}}_{{\mathrm{ox}}.{\mathrm{lim}}} - {\mathrm{f}}_{{\mathrm{eux}}} - {\mathrm{f}}_{{\mathrm{red}}}$$

The burial scaling factor, *α*, introduces a pseudospatial burial coefficient to the model, such that burial in redox-sensitive sinks is scaled to the anticipated effects of organic carbon remineralization, depending on the extent of euxinic and broadly reducing conditions across the continental shelf. Where z = water depth, b_oxic_ scales linearly such that:

$${\upalpha}_{{\mathrm{oxic}}} = 1$$

**Table 1:** Minimum and maximum values included in metal mass balances.

Variable	Minimum value	Maximum value
$\Delta ^{238}{\rm{U}}_{{\rm{eux}}}$ *‰*	0.4 ^20^	0.8 ^20^
$\Delta ^{238}{\rm{U}}_{{\rm{eux}}.{\rm{loc}}}$ *‰*	0.4 ^20^	0.8 ^20^
$\Delta ^{98}{\rm{Mo}}_{{\rm{eux}}}$ *‰*	−0.8 ^47^	0 ^31^
$\Delta ^{98}{\rm{Mo}}_{{\rm{eux}}.{\rm{loc}}}$* ‰*	−0.8 ^47^	0 ^31^
F_riv_(U) *mol yr*^−1^	2.75 × 10^7^ ^38^	5.65 × 10^7^ ^38^
F_riv_(Mo) *mol yr*^−1^	1.88 × 10^8^ ^75^ ^a^	3.0 × 10^8^ ^36^
b_U.eux_ *mol m*^−2^ *yr*^−1^	5.4 × 10^−6^ ^38^	4.62 × 10^−5^ ^38^
b_Mo.eux_ *mol m*^−2^ *yr*^−1^	5.0 × 10^−5^ ^36^ ^a^	1.25 × 10^−4^ ^75^ ^a^
b_U.red_ *mol m*^−2^ *yr*^−1^	9.2 × 10^−7^ ^38^ ^a^	4.37 × 10^−6^ ^38^ ^a^
b_Mo.red_ *mol m*^−2^ *yr*^−1^	2.61 × 10^−5^ ^75^ ^a^	2.81 × 10^−5^ ^36^ ^a^
b_U.ox_ *mol m*^−2^ *yr*^−1^	2.72 × 10^−8^ ^38^ ^a^	6.75 × 10^−8^ ^38^ ^a^
b_Mo.ox_ *mol m*^−2^ *yr*^−1^	2.08 × 10^−7^ ^75^ ^a^	2.87 × 10^−7^ ^36^ ^a^
b_U.eux.loc_ *mol m*^−2^ *yr*^−1^	5.4 × 10^−6^ ^38^	4.62 × 10^−5^ ^38^
b_Mo.eux.loc_ *mol m*^−2^ *yr*^−1^	5.00 × 10^−5^ ^36^ ^a^	1.25 × 10^−4^ ^75^ ^a^
δ^238^U_riv _*‰*	−0.40 ^76^ ^b^	−0.10 ^76^ ^b^
δ^98^Mo_riv_ *‰*	0.5 ^31^	0.9 ^31^
$\Delta ^{238}{\rm{U}}_{{\rm{red}}}$ *‰*	−0.23 ^40,77^ ^c^	0.23 ^44^
$\Delta ^{98}{\rm{Mo}}_{{\rm{red}}}$ *‰*	−2.8 ^78^	−0.8 ^31^
$\Delta ^{238}{\rm{U}}_{{\rm{oxic}}}$ *‰*	−0.043 ^79^	0.029 ^20^
$\Delta ^{98}{\rm{Mo}}_{{\rm{oxic}}}$ *‰*	−3.0 ^31^	−2.8 ^78^
$\Delta ^{238}{\rm{U}}_{{\rm{carb}}.{\rm{loc}}}$ *‰*	0.2 ^48^	0.4 ^48^
f_ox.lim_ *%*	83.89 ^36^	100 (physical limit)
f_red _*%*	0	100-f_eux_

^a^Calculated by unit conversion.

^b^Calculated as 5th and 95th percentile of ocean-draining rivers.

^c^References indicate that negative fractionations are possible under reducing conditions. As quantitative constraint on the magnitude of these fractionations is poor, the magnitude of the negative fractionation is parameterized to match the positive fractionation. See Supplementary Fig. 6 for alternative parameterizations.

Burial rates in euxinic settings scale with the expansion of euxinia, following the remineralization model^61^ used by Reinhard et al.^36^. In this scaling algorithm:

8 $${\upalpha}_{{\mathrm{eux}}} = \frac{{\mathop {\sum }\nolimits_{{\mathrm{min}}({\mathrm{z}}_{{\mathrm{eux}}})}^{{\mathrm{max}}({\mathrm{z}}_{{\mathrm{eux}}})} 1.58 - 0.16\ln \left( {{\mathrm{z}}_{{\mathrm{eux}}}} \right)}}{{{\mathrm{N}}({\mathrm{z}}_{{\mathrm{eux}}}){\upalpha}_{{\mathrm{eux}}.{\mathrm{min}}}}}$$

Where

$${\upalpha}_{{\mathrm{eux}}.{\mathrm{min}}} = 1.58 - 0.16\ln \left( {{\mathrm{min}}\left( {{\mathrm{z}}_{{\mathrm{eux}}}} \right)} \right)$$

and N(Z_eux_) describes the number of depths modeled, and following Reinhard et al.^36^ “we assume first that ∼5% of the shallow seafloor remains essentially authigenically neutral unless it becomes absolutely necessary to encroach upon this area (i.e., above 95% seafloor anoxia or euxinia)”. min(Z_eux_) and max(Z_eux_) are defined by a cross-validated LOESS model fitted to the global ocean depth-area relationships of Menard & Smith^62^ based on the approach of Reinhard et al.^36^

Burial rates in broadly reducing settings are also expected to scale with the extent of euxinic and broadly reducing conditions. Following previous application of Menard & Smith^62^ to metal mass balances^36^, broadly reducing (ferruginous + suboxic) settings are therefore modeled as expanding below the euxinic depositional environments along continental shelves in the pseudospatial scaling algorithm.

9 $${\upalpha}_{{\mathrm{red}}} = \frac{{\mathop {\sum }\nolimits_{{\mathrm{min}}({\mathrm{z}}_{{\mathrm{red}}})}^{{\mathrm{max}}({\mathrm{z}}_{{\mathrm{red}}})} 1.58 - 0.16\ln \left( {{\mathrm{z}}_{{\mathrm{red}}} + {\mathrm{max}}({\mathrm{z}}_{{\mathrm{eux}}})} \right)}}{{{\mathrm{N}}({\mathrm{z}}_{{\mathrm{red}}}){\upalpha}_{{\mathrm{eux}}.{\mathrm{min}}}}}$$

Where N(Z_red_) describes the number of depths modeled, min(Z_red_) is defined as max(Z_eux_), and max(Z_red_) is a function of A_red_+A_eux_, again defined by a cross-validated LOESS model fitted to the global ocean depth-area relationships of Menard & Smith^62^.

The effect of *α*_red _= 1 (no pseudospatial scaling in broadly reducing settings) is illustrated in Supplementary Fig. 6.

The purpose of this mass balance approach is to generate frequency distributions of feasible synthetic sedimentary Mo-U isotope values for a range of paleoredox scenarios. This is achieved by calculating euxinic shale and carbonate values as described in Eqs. 10–12, with fractionation factors in local settings allowed to vary independently of the global means.

10 $${\updelta}^{98}{\mathrm{Mo}}_{{\mathrm{eux}}} = {\updelta}^{98}{\mathrm{Mo}}_{{\mathrm{sw}}} + \Delta ^{98}{\mathrm{Mo}}_{{\mathrm{eux}}.{\mathrm{loc}}}$$

11 $${\updelta}^{238}{\mathrm{U}}_{{\mathrm{eux}}} = {\updelta}^{238}{\mathrm{U}}_{{\mathrm{sw}}} + \Delta ^{238}{\mathrm{U}}_{{\mathrm{eux}}.{\mathrm{loc}}}$$

12 $${\updelta}^{238}{\mathrm{U}}_{{\mathrm{carb}}} = {\updelta}^{238}{\mathrm{U}}_{{\mathrm{sw}}} + \Delta ^{238}{\mathrm{U}}_{{\mathrm{carb}}.{\mathrm{loc}}}$$

Mass balance equations are solved using a variable coefficient ordinary differential equation solver to fully couple marine trace metal concentrations and isotope values (rather than assuming instantaneous steady-state), and 1000 time-dependent Monte Carlo simulations are run to steady-state for 31 logarithmically scaled scenarios of ocean euxinia. Monte Carlo simulations are performed by global sensitivity analyses using the R packages deSolve^63^ and FME^64^ to subsample uniform prior distributions between prescribed minimum and maximum values. Minimum and maximum values for Monte Carlo variables are listed, with associated references, in Table 1. Fixed model parameters are listed with associated references in Table 2.

**Table 2:** Fixed values of modern oceanographic variables.

Variable	Value
Area of seafloor (A) *m*^2^	3.6 × 10^14^ ^36^
Mass of seawater (M) *kg*	1.41 × 10^21^ ^20^
[Mo]_M.sw_ *mol kg*^−1^	150 × 10^9^ ^37^
[U]_M.sw_ *mol kg*^−1^	14 × 10^9^ ^37^
δ^98^Mo_M.sw_ *‰*	2.34 ^31^
δ^238^U_M.sw_ *‰*	−0.39 ^80^

Time-dependent f_eux_ reconstructions in Fig. 3 are generated by fitting cross-validated LOESS models to percentiles of the f_eux_ distribution modeled for each timestep. In this case, cross-validated LOESS models are fitted to the δ^238^U_carb_ data from Bartlett et al.^8^ and the measured δ^98^Mo_eux_ and δ^238^U_eux_ data presented here, based upon their associated age models (see Stratigraphic age models). At 20 kyr timesteps between 444.7 and 440.8 Ma, ‘measured’ geochemical values are generated from these local regression models for each metal isotope proxy archive that (based on stratigraphic age models—see below) has recorded data for that time interval. The next step in this time-dependent modeling exercise is to describe modeled distributions of f_eux_ for each geological timestep. In order to preserve the resolution of distributions presented in Fig. 2, we binned both modeled and measured sedimentary geochemical data before filtering the modeled data set based on measured sedimentary values. Measured geochemical data for each proxy were subdivided into 10 proxy-specific bins (spaced uniformly between the minimum and maximum measured values). Modeled data were then binned at the same resolution for each of the three metal isotope proxy archives investigated. The binned f_eux_ distribution from global sensitivity analyses was then filtered to only include results compatible with measured geochemical data at each timestep in order to calculate the distributions described in Fig. 3a. Cross-validated LOESS models are used throughout this modeling workflow to reduce modeling subjectivity.

### Sample digestion and geochemical analyses

Shale samples were obtained from drill core and crushed to fine powder in a tungsten carbide shatterbox. Iron speciation measurements were performed at Stanford University following the methods of Poulton & Canfield^65^ for sequential extraction and Canfield et al.^66^ for chromium reduction of sulfur (CRS). Pyrite sulfur isotope values were analyzed on silver sulfide from the CRS extraction, measured on an Isoprime 100 isotope ratio mass spectrometer interfaced with an Elementar vario ISOTOPE cube elemental analyzer at Virginia Tech. Total organic carbon concentrations and carbon isotope values were first presented in Loydell et al.^25^, and were measured on a Finnegan Mat 251 mass spectrometer coupled to a Fisons 1108 elemental analyzer. Major and trace metal concentrations were measured at Bureau Veritas/ACME Labs using standard four acid digestion techniques and Q-ICP-MS/ICP-OES.

Uranium and molybdenum isotopes were measured at the Yale Metal Geochemistry Center using previously described acid digestion, column chromatography, and MC-ICP-MS methods^44,67,68^: All powdered samples were first ashed at 600 °C to remove organic matter, then dissolved at 105 °C in a concentrated HF-HNO_3_ mixture followed by an aqua regia digestion of the dried residues. Final residues were dissolved in 5 ml 6 N HCl as stock solutions. Splits from these solutions were then used for Mo and U column chromatography and subsequent analyses via MC-ICP-MS. Bulk trace metal concentrations and isotope measurements were corrected for detrital input with associated error estimates for authigenic values^69^ (Methods—Detrital corrections).

### Uranium isotope analyses

Uranium was separated using UTEVA ion exchange resin and the ^233^U–^236^U double spike method (see methods of Weyer et al.^44^ and Wang et al.^67^). Double spike was added to samples prior to column chemistry to achieve a ^236^U/^238^U ratio of ~30 based on previously measured U concentrations. Samples were then evaporated to dryness, brought up in 3 N HNO_3,_ and then purified on UTEVA resin columns using the method of Wang et al.^67^ with 100 ng loaded on the columns. After purification, U samples were dissolved in 0.3 N HNO_3_ to achieve 50 ppb solutions for analysis. Samples were measured on a Thermo-Finnigan Neptune Plus multi-collector ICP-MS. Samples were introduced using an Apex IR sample introduction system and measured at low resolution. Isotopes ^232^Th, ^233^U, ^235^U, ^236^U, and ^238^U were measured simultaneously on Faraday collectors connected to 10^11^ Ω amplifiers achieving a signal of ~ 40–45 volts on ^238^U.

The CRM 112a (New Brunswick Laboratory, US Department of Energy) standard was analyzed every three samples to monitor drift in instrumental mass bias, and samples were normalized to the average of bracketing standards. The drift of the standard was typically <0.08‰. Blank levels were always <0.1% of sample voltage, and no blank correction was made (the correction would be smaller than the instrumental counting error). External reproducibility was 0.1‰ based on duplicate sample preparations and analyses of both the USGS geostandard NOD-A-1 (0.60‰, 2 SD = 0.04‰, *n* = 4) and sample duplicates (*n *= 5). Values for U isotope measurements and associated error can be found in Supplementary Data 1.

### Molybdenum isotope analyses

Molybdenum isotope measurements were also performed at the Yale Metal Geochemistry Center following methods in Planavsky et al.^68^. An aliquot of the bulk digests was doped with a ^97^Mo–^100^Mo double spike, prepared gravimetrically from Oak Ridge Laboratory metal powders^68^. The spiked sample was dried to ensure sample-spike equilibration. We used a two-stage column separation^68^. We first ran the sample through an anion resin (AG-MP-1M) and then a cation resin (AG50W-X8) to remove any remaining Fe. The Mo isotopic ratios were analyzed using a Neptune Plus MC-ICP-MS. Molybdenum isotope compositions are reported using the δ notation, where δ^98/95^Mo (‰) = 1000 ∙ [(^98^Mo/^95^Mo)_sample_/(^98^Mo/^95^Mo)_NIST3134*0.99975_ − 1], calculated relative to NIST 3134 (Lot 130418) with a value of +0.25 ‰^70^. A calibration of the NIST standard relative to Rochester (Lot 862309E) gave: δ^98^Mo_ROCH_ = δ^98^Mo_NIST3134_ − 0.32 ± 0.12 ‰. For each sample, the target Mo concentration was 50 ppb during each session. In all reported samples the 2 SD was <0.1 ‰. Duplicates (*n* = 4) of reference standard USGS NOD-P-1 had an average δ^98^Mo value of −0.67‰ and 2 SD of 0.03 ‰. Values for Mo isotope measurements and associated error can be found in Supplementary Data 1.

### Detrital corrections

Detrital corrections are made for bulk molybdenum and uranium isotope and concentration measurements to infer authigenic isotope and concentration values. Authigenic molybdenum estimates are generally very similar to bulk measured values, as expected based on understanding of detrital trace metal shuttling^45^. However, both metals are standardized for consistency. Uranium values are standardized to thorium based on the conservative behavior demonstrated in Cole et al.^69^; molybdenum values are standardized to aluminum based on the methods of Noordmann et al.^71^, using crustal average data from Rudnick & Gao^72^. Standardizing uranium to aluminum results in lighter δ^238^U than standardizing to thorium (corresponding to more widespread euxinia; Supplementary Data 1), so thorium standardization is presented as the most conservative estimate of authigenic δ^238^U.

$$[{\mathrm{Mo}}]_{{\mathrm{detr}}} = \frac{{[{\mathrm{Mo}}]_{{\mathrm{crust}}}}}{{[{\mathrm{Al}}]_{{\mathrm{crust}}}}}[{\mathrm{Al}}]_{{\mathrm{bulk}}}$$

$$[{\mathrm{U}}]_{{\mathrm{detr}}} = \frac{{[{\mathrm{U}}]_{{\mathrm{crust}}}}}{{[{\mathrm{Th}}]_{{\mathrm{crust}}}}}[{\mathrm{Th}}]_{{\mathrm{bulk}}}$$

$$[{\mathrm{Mo}}]_{{\mathrm{auth}}} = [{\mathrm{Mo}}]_{{\mathrm{bulk}}} - [{\mathrm{Mo}}]_{{\mathrm{detr}}}$$

$$[{\mathrm{U}}]_{{\mathrm{auth}}} = [{\mathrm{U}}]_{{\mathrm{bulk}}} - [{\mathrm{U}}]_{{\mathrm{detr}}}$$

$${\mathrm{\delta }}^{98}{\mathrm{Mo}}_{{\mathrm{auth}}} = \frac{{{\mathrm{\delta }}^{98}{\mathrm{Mo}}_{{\mathrm{bulk}}}[{\mathrm{Mo}}]_{{\mathrm{bulk}}} - {\mathrm{\delta }}^{98}{\mathrm{Mo}}_{{\mathrm{detr}}}[{\mathrm{Mo}}]_{{\mathrm{detr}}}}}{{[{\mathrm{Mo}}]_{{\mathrm{auth}}}}}$$

$${\mathrm{\delta }}^{238}{\mathrm{U}}_{{\mathrm{auth}}} = \frac{{{\mathrm{\delta }}^{238}{\mathrm{U}}_{{\mathrm{bulk}}}[{\mathrm{U}}]_{{\mathrm{bulk}}} - {\mathrm{\delta }}^{238}{\mathrm{U}}_{{\mathrm{detr}}}[{\mathrm{U}}]_{{\mathrm{detr}}}}}{{[{\mathrm{U}}]_{{\mathrm{auth}}}}}$$

With modern crustal measurements of δ^98^Mo_detr_ from Voegelin et al.^73^ and δ^238^U_detr_ from Weyer et al.^44^ Error estimates on detrital concentrations (confidence intervals recommended in Cole et al.^69^; 2 SD from Cole et al.^69^ and Rudnick & Gao^72^, for U and Mo, respectively) are combined with analytical errors (2 SE) to generate uncertainties on metal concentration and isotope values, as seen in Fig. 1, and Supplementary Figs. 3–5 (Supplementary Data 1).

### Stratigraphic age models

Extrapolating the time-independent models of Fig. 2 to the time-dependent biogeochemical change modeled in Fig. 3a is dependent on stratigraphic age models. For the E1-NC174 core (data measured in this study), we use a continuous stratigraphic age model beginning at the Hirnantian-Rhuddanian boundary (443.8 Ma) and ending at the Rhuddanian-Aeronian boundary (440.8 Ma). Although the core does not definitively contain these boundaries based on biostratigraphy, the core does contain all graptolite biozones of the Rhuddanian^21^ and this is therefore proposed as the most appropriate age model. For the published δ^238^U_carb_ data, we use a continuous stratigraphic age model based on the description in Bartlett et al.^8^ and references therein (including Wickson^74^). The age models used in both Finnegan et al. studies^1,5^ presented in Fig. 3 are updated to reflect the Geologic Timescale 2012^23^ and illustrate stratigraphic relationships relative to δ^238^U_carb_ data^8^ where applicable. Notes justifying the placement of placement of stratigraphic boundaries in these stratigraphic age models are included in Supplementary Data 2.

### Reporting summary

Further information on research design is available in the Nature Research Reporting Summary linked to this article.

## Supplementary information

Supplementary Information

Description of Additional Supplementary Files

Supplementary Data 1

Supplementary Data 2

Reporting Summary

## Data Availability

The data presented in this manuscript are included in Supplementary Data File 1 (all new geochemical data presented in Fig. 1 and Supplementary Figs. 2–5) and Supplementary Data File 2 (data from published studies used in Fig. 3 with revised age models and associated justifications).

## Code availability

The model code required for the analyses presented in this manuscript can be freely accessed on Github [https://github.com/richardstockey/Mo.U.Model].
