# Supplementary material for: Persistent global marine euxinia in the early Silurian
Source: Nat Commun. 2020 Apr 14;11:1804. doi: 10.1038/s41467-020-15400-y (PMC7156380; doi:10.1038/s41467-020-15400-y)
Supplement: Supplementary file 1 — Supplementary Information [file 41467_2020_15400_MOESM1_ESM.pdf]

Supplementary Information

Persistent global marine euxinia in the early Silurian

Stockey et al.

Supplementary Note 1: Additional geological background

The Tannezuft Shale Formation unconformably overlies the Late Ordovician Mamuniyat Formation and includes the mid-Rhuddanian 'hot shale' source rock that has motivated its extensive biostratigraphic characterization. Graptolite[1], acritarch[2] and chitinozoan[3] biostratigraphy all indicate that the E1-NC174 core is Rhuddanian in age. The lowest strata in the core contain *Normalograptus tilokensis*, a graptolite species known primarily from the Upper Ordovician but now believed to have persisted into the early Silurian. It is therefore ambiguous whether the cored interval contains the Ordovician-Silurian boundary, but E1-NC174 at least contains all graptolite biozones of the Rhuddanian Age. A continuous sedimentation model, assuming that the cored interval represents the entire Rhuddanian Age, was used for time-dependent reconstructions of f_{eux} through the Rhuddanian. This depositional model, and the stratigraphic age model used in Bartlett et al.[4] (replicated here based on their description and supporting references), are reliant on Rhuddanian age estimates from the Geologic Timescale 2012[5] and simplistic assumptions of continuous sedimentation rates. We view this age model as the most appropriate for the illustration of results in Figures 2-3 and Supplementary Figures 5-6 based on current knowledge of Rhuddanian stratigraphy.

The E1-NC174 core comprises organic-rich, laminated dark grey shales, with the mid-Rhuddanian 'hot shale' interval defined by increased total organic carbon and spectral gamma ray values[2]. The general absence of bioturbation has previously been interpreted as indicating bottom-water anoxia, with two recorded examples of minor (probably chondritiform) burrowing[2] requiring only transient local increases in dissolved oxygen at shorter timescales than those preserved in the geochemical record. Rock-Eval analyses of the 'hot shale' interval in other parts of the Murzuq Basin demonstrate that these organic-rich mid-Rhuddanian shales contain abundant immature to early mature type II/III kerogen[6] (approx. 400-450°C T_{max}). Core samples were originally logged in feet and inches; depths have been independently converted to meters for the purpose of this study.

Supplementary Note 2: Paleobiological context for the Late Ordovician mass extinction

The per-capita brachiopod extinction rates illustrated in Figure 3B are replotted from Finnegan et al.[7] using R code available on GitHub (https://github.com/sethfinnegan/Ord.Brach.Extinctions.Code). The age model used in Finnegan et al.[7] was updated here to illustrate the timing of extinction pulses relative to the Geologic Timescale 2012[5]. The spacing of these data within geological periods is consistent with the original study (Supplementary Data 2). Per lineage million year graptoloid extinction rates illustrated in Figure 3B are replotted from Crampton et al[8,9]. The two globally recorded extinction pulses of the Late Ordovician mass extinction are annotated on Fig. 3 based on Kröger et al.[10]. Capture-recapture analyses of genus diversity through the Cambrian-Silurian were rerun using R code from Rasmussen et al.[11] and plotted in Figure 3D.

Supplementary Note 3: Ordovician-Silurian climate

Clumped isotope data from Finnegan et al.[12] are replotted in Fig. 3C. The line describing the data is fitted to the minimum temperature estimates for each time bin and is intended to match the interpretations of the original publication. The age model used by Finnegan et al.[12] is updated to include both the ages described in the Geologic Timescale 2012[5] and the stratigraphic relationships for Anticosti Island described in Bartlett et al.[4]. This presentation of the results therefore differs from the original study but is intended to provide the most useful temporal comparison with the trace metal isotope modeling presented in Fig. 3A. The same filtering methods to select samples subject to minimal diagenetic alteration are applied as in the original study. Reconstructions of peak Hirnantian glaciation followed by rapid late Hirnantian/Rhuddanian warming are further supported by phosphate oxygen isotope data[4] and inferences from the sedimentary record[13–15]. Climatic reconstructions of the Ordovician-Silurian Earth system commonly invoke elevated atmospheric pCO_2 levels relative to the modern atmosphere, with Rhuddanian pCO_2 estimates reaching approximately 8 times present atmospheric levels[15]. We illustrate the implications of these atmospheric reconstructions on weathering rates, and by extension metal fluxes, in Scenario 5 of Supplementary Figure 6. In these calculations, the maximum input fluxes from Table 1 are multiplied by a coefficient of $8^{0.22}$ based on Berner et al.[16], with 8 PAL representing a minimum estimate for Rhuddanian CO_2[15] and

0.22 a fixed scaling factor. As shown in Supplementary Figure 6, these increased weathering fluxes have little impact on our model results.

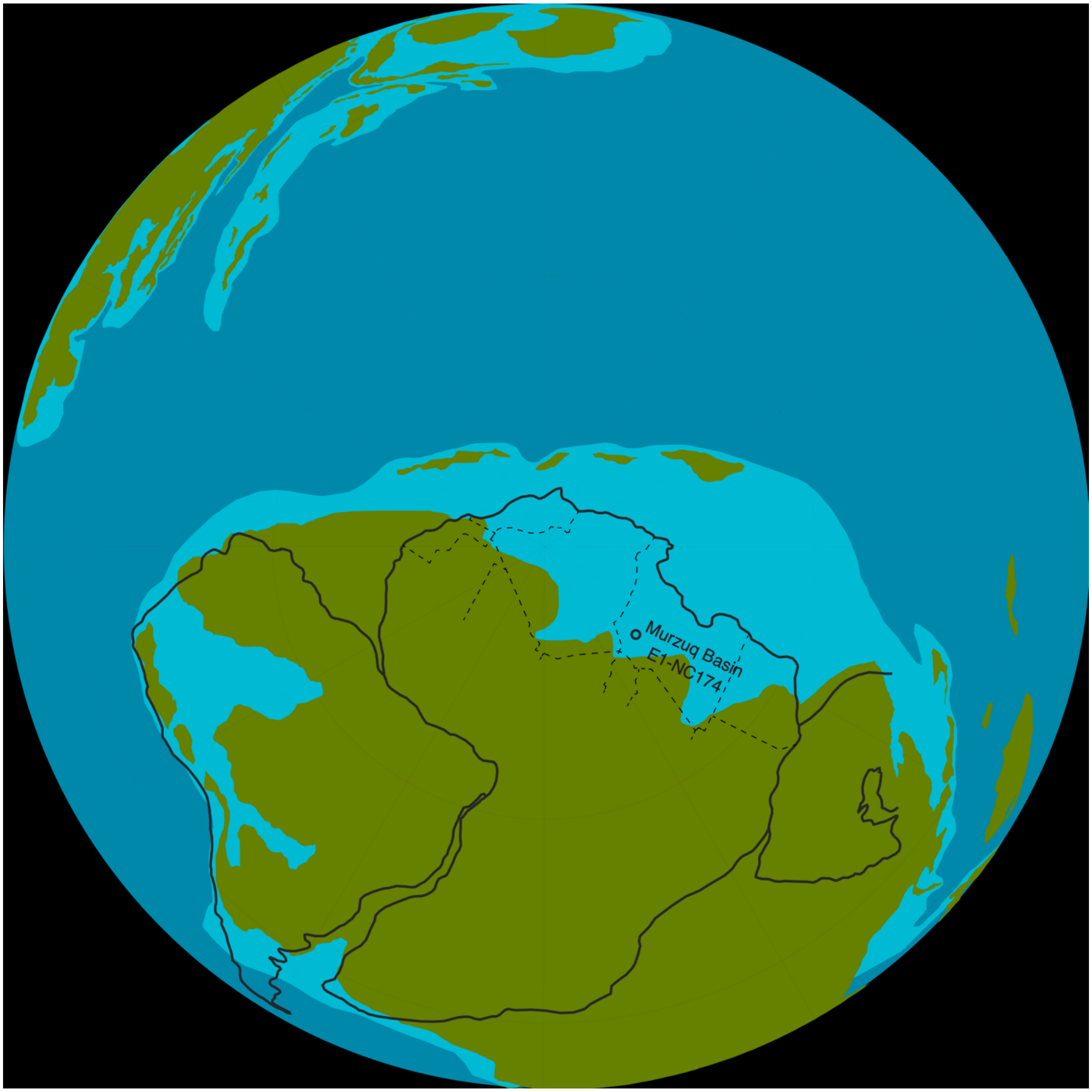

Supplementary Figure 1: Silurian tectonic reconstruction based on Blakey[17], projected from the South Pole, with the approximate location of the Murzuq Basin and E1-NC174 core annotated based on Loydell et al.[2].

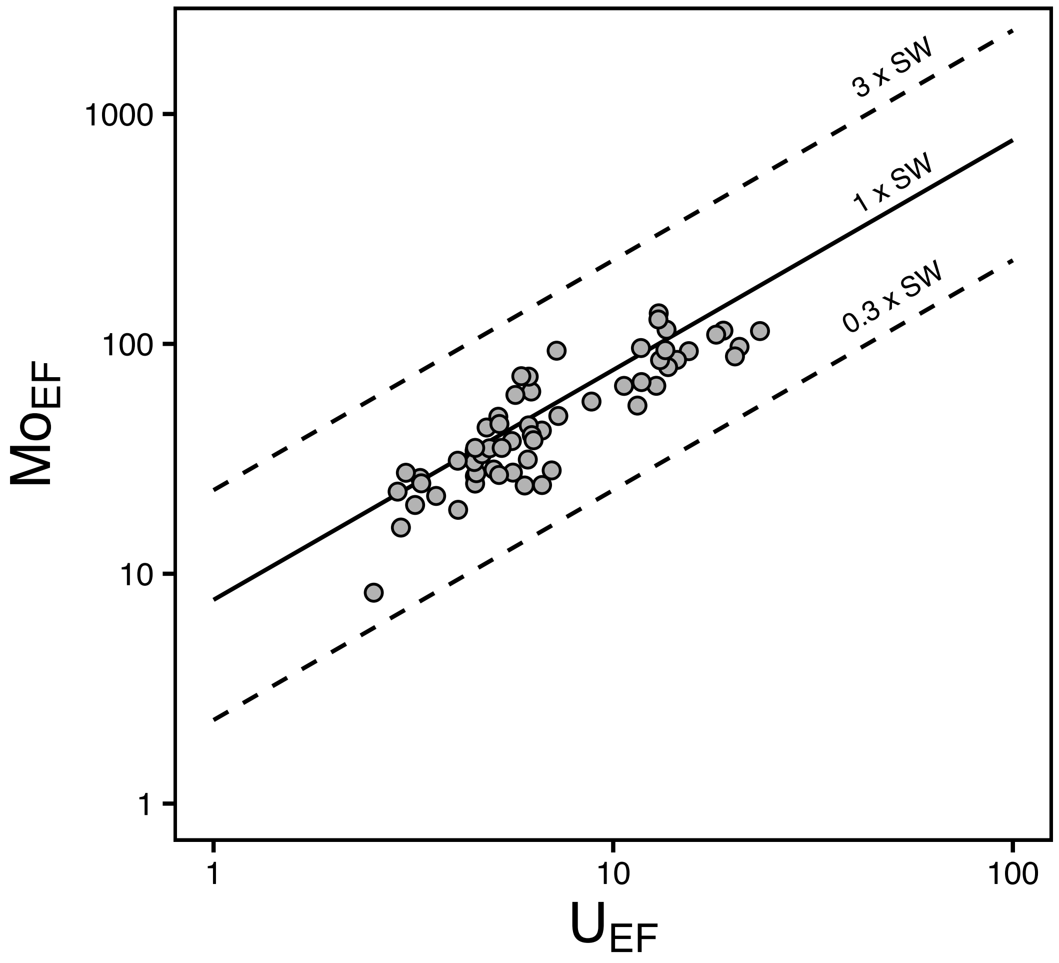

Supplementary Figure 2: Correlations between molybdenum and uranium enrichment factors for E1-NC174 samples described in this study. Lines describe seawater Mo-U ratios based on Algeo & Tribovillard[20], fine-grained sediments deposited in the presence of a significant particulate shuttle are expected to plot above the 3 x SW line (3-10 times seawater Mo-U ratios[20]).

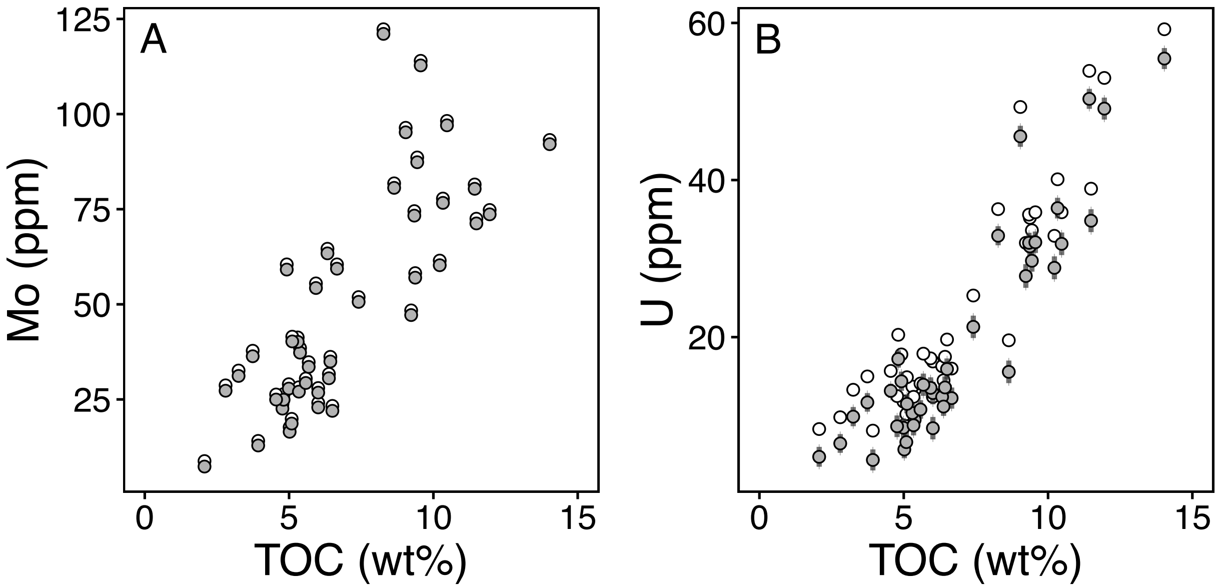

Supplementary Figure 3: Correlations between redox sensitive metal and total organic carbon (TOC) concentrations for the E1-NC174 samples described in this study. A) Molybdenum vs TOC. B) Uranium vs TOC.

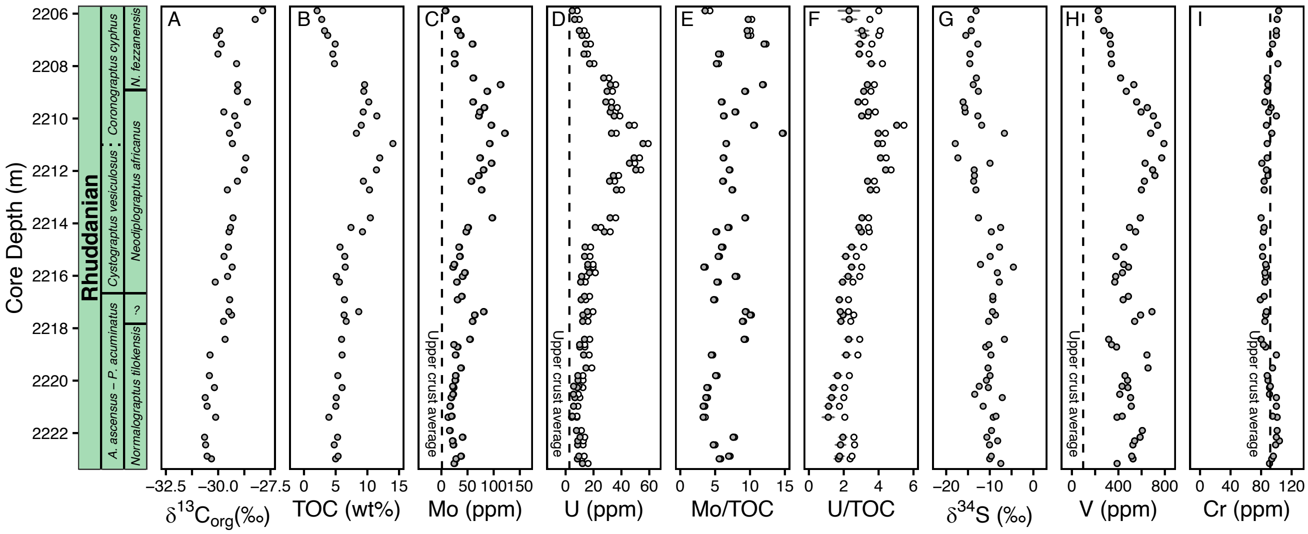

Supplementary Figure 4: Additional geochemistry of the E1-NC174 core. Core depth is presented alongside the global and regional Rhuddanian graptolite biozones based on Loydell[1,18]. A) Organic carbon isotope values are presented from Loydell et al.[2]. B) Total organic carbon concentrations (TOC) are presented from Loydell et al.[2]. C) Mo concentrations are expressed in ppm, relative to average crustal values from Rudnick & Gao[19]. D) U concentrations are expressed in ppm, relative to average crustal values from Rudnick & Gao[19]. E) Mo concentrations are standardized to total organic carbon concentration (ppm/wt%), as in Fig. 1. F) U concentrations are standardized to total organic carbon concentration (ppm/wt%), as in Fig. 1. G) Pyrite sulfur isotope ($\delta^{34}S$) values. H) V concentrations, relative to average crustal values from Rudnick & Gao[19]. I) Cr concentrations, relative to average crustal values from Rudnick & Gao[19]. As in Figure 1, in Mo and U concentration plots, white points represent bulk measurements, and grey points represent measurements corrected for detrital input, with associated error (for molybdenum, the difference between these values is often negligible). Error bars associated with Mo and U concentrations illustrate 2SD uncertainty on crustal concentrations used in detrital corrections.

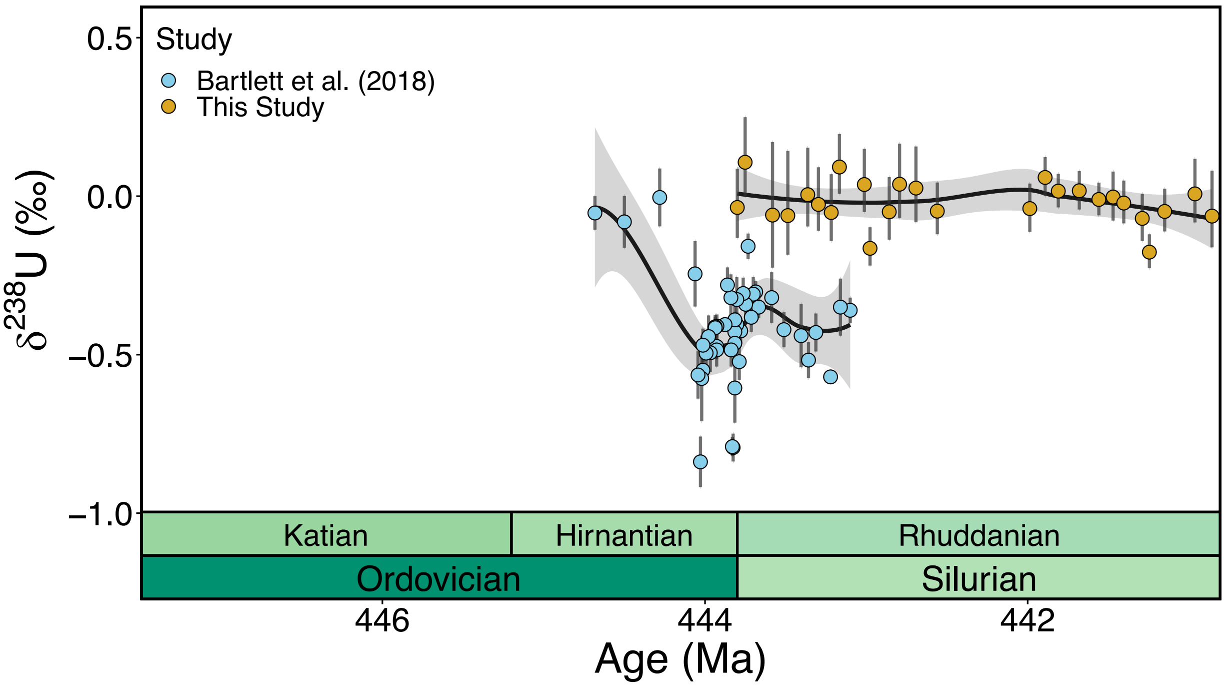

Supplementary Figure 5: All uranium isotope data (and associated stratigraphic age models presented in this study). Bartlett et al.[4] data are from shallow-water carbonates, data in this study are from euxinic shales. An offset of ~0.5 ‰ is observed between Rhuddanian carbonates and shales, consistent with a ~0.8 ‰ fractionation between euxinic shales and contemporaneous seawater[21,22]. Error bars associated with trace metal isotopes illustrate combined analytical error (2SE) and 2SD uncertainty on crustal concentrations used in detrital corrections.

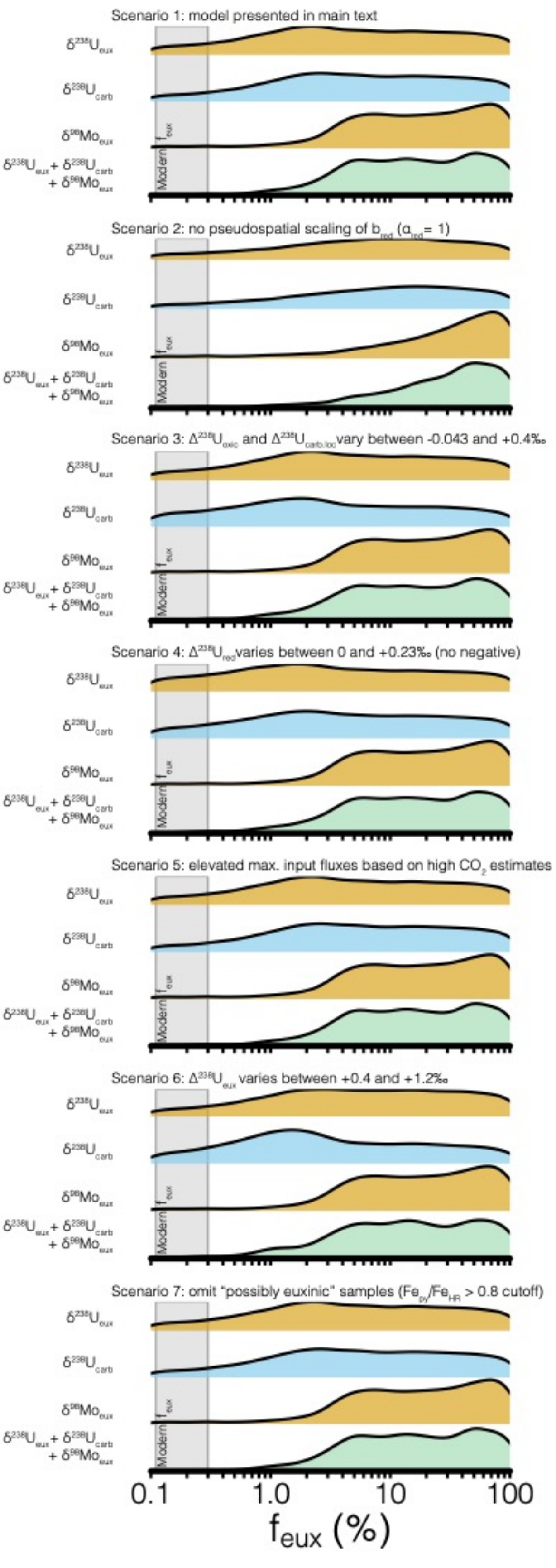
Scenario 1: model presented in main text
Scenario 2: no pseudospatial scaling of b_red (α_red = 1)
Scenario 3: Δ238U_oxic and Δ238U_carb.loc vary between -0.043 and +0.4‰
Scenario 4: Δ238U_red varies between 0 and +0.23‰ (no negative)
Scenario 5: elevated max. input fluxes based on high CO2 estimates
Scenario 6: Δ238U_eux varies between +0.4 and +1.2‰
Scenario 7: omit "possibly euxinic" samples (Fe_py/Fe_HR > 0.8 cutoff)
δ238U_eux
δ238U_carb
δ98Mo_eux
δ238U_eux + δ238U_carb + δ98Mo_eux
Modern f_eux
0.1
1.0
10
100
f_eux (%)

Supplementary Figure 6: Alternative Monte Carlo parameterizations of key model variables. For each scenario, smoothed density distributions represent the distributions of f_{eux} scenarios compatible with the measured $\delta^{238}U_{eux}$, $\delta^{238}U_{carb}$[4] ,$\delta^{98}Mo_{eux}$, and combined carbonate[4] and euxinic shale Mo-U isotope data ($\delta^{238}U_{eux}$ + $\delta^{238}U_{carb}$ + $\delta^{98}Mo_{eux}$) (labelled, from top to bottom). The fully coupled carbonate and euxinic shale Mo and U isotope distribution is taken as the best-constrained model of f_{eux} through the Rhuddanian. Specifics of alternative scenarios are annotated on each boxplot (Scenario 5 is described in full in Supplementary Note 3).

Supplementary References

1. Loydell, D. K. Graptolite biostratigraphy of the E1-NC174 core, Rhuddanian (lower Llandovery, Silurian), Murzuq Basin (Libya). *Bull. Geosci.* **84**, 651–660 (2011).
2. Loydell, D. K., Butcher, A. & Frýda, J. The middle Rhuddanian (lower Silurian) 'hot' shale of North Africa and Arabia: An atypical hydrocarbon source rock. *Palaeogeogr. Palaeoclimatol. Palaeoecol.* **386**, 233–256 (2013).
3. Butcher, A. Chitinozoans from the middle Rhuddanian (lower Llandovery, Silurian) 'hot' shale in the E1-NC174 core, Murzuq Basin, SW Libya. *Rev. Palaeobot. Palynol.* **198**, 62–91 (2013).
4. Bartlett, R. *et al.* Abrupt global-ocean anoxia during the Late Ordovician–early Silurian detected using uranium isotopes of marine carbonates. *Proc. Natl. Acad. Sci. U. S. A.* **115**, 5896–5901 (2018).
5. Gradstein, F. M. *et al. The Geologic Time Scale 2012*. *Elsevier* **1**, (2012).
6. El Diasty, W. S., El Beialy, S. Y., Anwari, T. A. & Batten, D. J. Hydrocarbon source potential of the Tanezzuft Formation, Murzuq Basin, south-west Libya: An organic geochemical approach. *J. African Earth Sci.* **130**, 102–109 (2017).
7. Finnegan, S., Rasmussen, C. M. Ø. & Harper, D. A. T. Biogeographic and bathymetric determinants of brachiopod extinction and survival during the Late Ordovician mass extinction. *Proc. R. Soc. B Biol. Sci.* **283**, 20160007 (2016).
8. Crampton, J. S., Cooper, R. A., Sadler, P. M. & Foote, M. Greenhouse-icehouse transition in the Late Ordovician marks a step change in extinction regime in the marine plankton. *Proc. Natl. Acad. Sci. U. S. A.* **113**, 1498–503 (2016).
9. Crampton, J. S. *et al.* Pacing of Paleozoic macroevolutionary rates by Milankovitch grand cycles. *Proc. Natl. Acad. Sci.* **115**, 5686–5691 (2018).
10. Kröger, B., Franeck, F. & Rasmussen, C. M. Ø. The evolutionary dynamics of the early Palaeozoic marine biodiversity accumulation. *Proc. R. Soc. B Biol. Sci.* **286**, (2019).
11. Rasmussen, C. M. Ø., Kröger, B., Nielsen, M. L. & Colmenar, J. Cascading trend of Early Paleozoic marine radiations paused by Late Ordovician extinctions. *Proc. Natl. Acad. Sci. U. S. A.* **116**, 7207–7213 (2019).
12. Finnegan, S. *et al.* The magnitude and duration of Late Ordovician-Early Silurian glaciation. *Science* **331**, 903–6 (2011).
13. Melchin, M. J., Mitchell, C. E., Holmden, C. & Storch, P. Environmental changes in the Late Ordovician-early Silurian: Review and new insights from black shales and nitrogen isotopes. *Geol. Soc. Am. Bull.* **125**, 1635–1670 (2013).
14. Page, A., Williams, M. & Zalasiewicz, J. Were transgressive black shales a negative feedback mechanism modulating glacio-eustatic cycles in the Early Palaeozoic Icehouse? *Micropalaeontological Soc. Spec. Publ. Geol. Soc. London* **8**, 123–156 (2007).
15. Pohl, A., Donnadieu, Y., Le Hir, G. & Ferreira, D. The climatic significance of Late Ordovician-early Silurian black shales. *Paleoceanography* **32**, 397–423 (2017).
16. Berner, R. A. & Kothavala, Z. Geocarb III: A revised model of atmospheric CO2 over phanerozoic time. *Am. J. Sci.* **301**, 182–204 (2001).
17. Blakey, R. *Global Paleogeography and Tectonics in Deep Time*. (Colorado Plateau Geosystems Inc., 2016).
18. Loydell, D. K. Graptolite biozone correlation charts. *Geol. Mag.* **149**, 124–132 (2012).
19. Rudnick, R. L. & Gao, S. Composition of the Continental Crust. in *Treatise on Geochemistry: Second Edition* **4**, 1–51 (Elsevier, 2014).
20. Algeo, T. J. & Tribovillard, N. Environmental analysis of paleoceanographic systems based on molybdenum-uranium covariation. *Chem. Geol.* **268**, 211–225 (2009).
21. Weyer, S. *et al.* Natural fractionation of $^{238}U/^{235}U$. *Geochim. Cosmochim. Acta* **72**, 345–359 (2008).
22. Romaniello, S. J., Herrmann, A. D. & Anbar, A. D. Uranium concentrations and 238U/235U isotope ratios in modern carbonates from the Bahamas: Assessing a novel paleoredox proxy. *Chem. Geol.* **362**, 305–316 (2013).
