# Supplementary material for: Persistent global marine euxinia in the early Silurian
Source: Nat Commun. 2020 Apr 14;11:1804. doi: 10.1038/s41467-020-15400-y (PMC7156380; doi:10.1038/s41467-020-15400-y)
Supplement: Supplementary file 2 — Description of Additional Supplementary Files [file 41467_2020_15400_MOESM2_ESM.pdf]

Description of Additional Supplementary Files

File Name: Supplementary Data 1
Description: All sedimentary geochemical data from the E1-NC174 core (Murzuq Basin, Libya) presented in this study, with associated errors, core depths and modeled ages.

File Name: Supplementary Data 2
Description: Published geochemical data and extinction rate data for which stratigraphic age models were developed in this study, with associated age justifications.
